# Management of Injury to the Common Bile Duct in a Patient with Roux-en-Y Gastric Bypass

**DOI:** 10.1155/2014/938532

**Published:** 2014-08-05

**Authors:** Sheraz Yaqub, Tom Mala, Øystein Mathisen, Bjørn Edwin, Bjarte Fosby, Dag Tallak Kjærsdalen Berntzen, Andreas Abildgaard, Knut Jørgen Labori

**Affiliations:** ^1^Department of Hepato-Pancreato-Biliary Surgery, Oslo University Hospital, Sognsvannsveien 20, 0317 Oslo, Norway; ^2^Department of Gastrointestinal Surgery, Oslo University Hospital, Sognsvannsveien 20, 0317 Oslo, Norway; ^3^Department of Transplantation Surgery, Oslo University Hospital, Sognsvannsveien 20, 0317 Oslo, Norway; ^4^Department of Radiology, Oslo University Hospital, Sognsvannsveien 20, 0317 Oslo, Norway

## Abstract

*Introduction*. Most surgeons prefer Roux-en-Y hepaticojejunostomy (RYHJ) for biliary reconstruction following a common bile duct (CBD) injury. However, in patients with a Roux-en-Y gastric bypass (RYGB) a RYHJ may be technically challenging and can interfere with bowel physiology induced by RYGB. The use of a hepaticoduodenostomy (HD) resolves both these issues. *Presentation of Case*. We present a case of CBD injury during laparoscopic cholecystectomy one year after laparoscopic RYGB for morbid obesity. Due to adhesions and previous surgery with RYGB, we did not want to interfere with the RYGB physiology by anastomosing the CBD to the jejunum or ileum. Succeeding a full Kocher's maneuver we performed biliary reconstruction by a tension-free end-to-side HD. The postoperative recovery was uneventful and the patient was discharged after eight days. At four-month follow-up, the patient had stable weight and normal laboratory test results. MRCP demonstrated normal intra- and extrahepatic bile ducts with status after HD. *Discussion*. We propose that HD should be considered in treatment of CBD injury in post-RYGB patients as it may reduce the risk of interfering with the post-RYGB physiology.

## 1. Introduction

After the introduction of laparoscopic cholecystectomy the management of bile duct injuries received increased attention [1–3]. The wave of surgery for morbid obesity (bariatric surgery) has developed a new patient cohort susceptible to gallbladder disease requiring cholecystectomy with the subsequent risk of bile duct injury. Different surgical methods are used for treatment of morbid obesity [4]. Laparoscopic Roux-en-Y gastric bypass (RYGB) is among the most common procedures. About 25–30% of the patients may develop gallbladder disease following bariatric surgery [5, 6]. An increase in laparoscopically induced bile duct injuries may be a consequence of an increasing number of cholecystectomies following RYGB. Cholecystectomy in such patients can be challenging due to the altered bowel anatomy, adhesions, and often the presence of an already constructed Roux-en-Y loop. Repair of bile duct injuries in such patients should in addition not interfere with the weight losing effect of the previous procedure. We demonstrate herein the first case, to our knowledge, of a patient with injury of the bile duct one year after RYGB for morbid obesity.

## 2. Case Presentation

A 32-year-old man was referred to our hospital because of common bile duct (CBD) injury during laparoscopic cholecystectomy at a local hospital. His medical history included laparoscopic RYGB for morbid obesity with a BMI 47 kg/m^2^. The operation was performed in East Europe and despite considerable efforts no operation record could be retrieved. One year after the operation, he presented with symptomatic cholecystolithiasis. He had no history of cholecystitis or cholangitis. During laparoscopic cholecystectomy intra-abdominal adherences were found which made the procedure more difficult and may have contributed to the transection of the CBD. As often is the case the injury was not appreciated immediately [2]. The patient developed jaundice and on postoperative day six a diagnostic laparoscopy revealed a transection of the CBD. He was immediately referred to our clinic, which is a tertiary care centre for HPB surgery.

**Figure 1:**
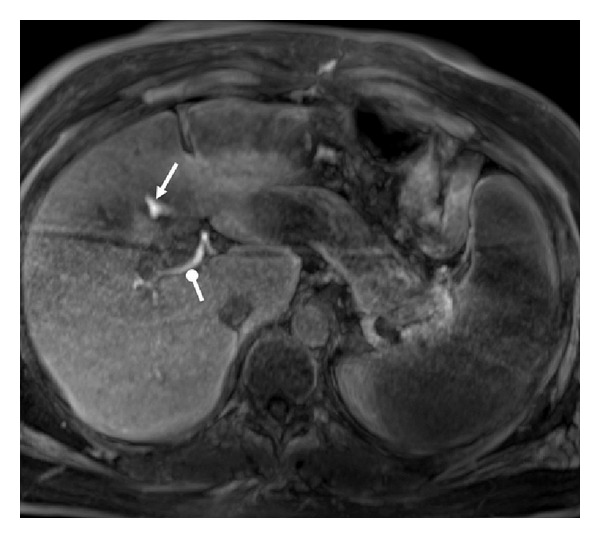
*Magnetic resonance imaging showing bile leakage.* Subvolume rendering of T1-weighted fat suppressed axial gradient echo acquisition approximately 45 minutes after intravenous administration of Gd-EOB-DTPA (Primovist; Bayer-Schering, Berlin, Germany) showing contrast-enhanced bile in central intrahepatic bile ducts (dot-head) and verifying bile leakage in gallbladder fossa (arrow).

Physical examination disclosed no abnormalities except for mild abdominal tenderness in right upper quadrant and a mild jaundice. BMI was 31 kg/m^2^. Blood analyses showed CRP 9 mg/L and bilirubin 97 *μ*mol/L; other laboratory studies were normal. Contrast-enhanced multislice computed tomography (CT) confirmed the presence of moderate water-density free fluid in the abdomen, but no free peritoneal air. Bile duct dilatation or vascular injuries were not observed. Magnetic resonance imaging (MRI) using the liver-specific contrast agent gadoxetic acid (Gd-EOB-DTPA, Primovist, Bayer-Schering, Berlin, Germany) [7] depicted transection of the CBD and pathological extrahepatic accumulation of contrast medium in the hepatobiliary phase scan (45 minutes after contrast medium injection) confirming the diagnosis of postoperative bile leakage (Figure 1).

**Figure 2:**
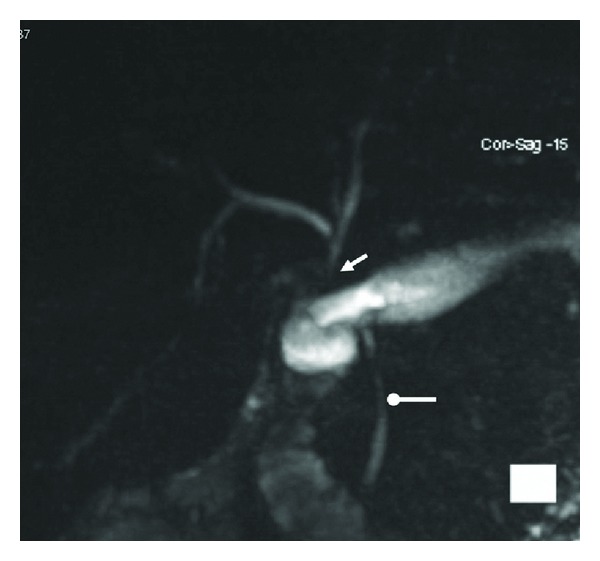
*MRCP showing hepaticoduodenostomy.* Three-dimensional rendering (right anterior oblique projection) of thin-slice MRCP (magnetic resonance cholangiopancreatography) showing normal size of intrahepatic bile ducts and a short hepatic duct with hepaticoduodenostomy (arrow). Native choledochal duct is also seen (dot-head).

The patient was operated with a midline laparotomy with a right-sided subcostal extension. We identified the first operation as a RYGB. The transected CBD was identified and classified as Strasberg E2 [8]. The diameter of the CBD was approximately 5 mm. As the outcome of the RYGB had been successful we did not want to interfere with the Roux-en-Y loop and decided to perform an end-to-side hepaticoduodenostomy (HD). This was done using a running 5-0 Prolene suture, after a full Kocher's maneuver to gain satisfactory mobilization to make the anastomosis tension-free. The postoperative recovery was uneventful and the patient was discharged after eight days. A four-month follow-up revealed normal physical examination. The patient had stable weight with a BMI of 31 kg/m^2^ and normal lab tests. MRI showed normal intra- and extrahepatic bile ducts with a status after HD (Figure 2).

## 3. Discussion 

Bile duct injury after elective laparoscopic cholecystectomy has been reported to range from 0.2% to 0.7% [1, 9]. The surgical approach to morbid obesity has gained wide acceptance in recent years. The risk of cholelithiasis is increased in these patients and an increased incidence of bile duct injuries would not be surprising. However, the incidence of bile duct injuries during laparoscopic cholecystectomy after previous bariatric surgery is unknown.

To avoid a second procedure due to complications from gallbladder disease, studies have looked into the benefit of concomitant cholecystectomy during the operation for morbid obesity. A recent meta-analysis recommends that prophylactic cholecystectomy during RYGB should only be performed in patients with symptomatic biliary disease [10]. However, it should be considered that a second procedure could be technically more demanding with longer operating time, higher conversion rate, and the risk for bile duct and vascular injuries.

Surgical repair of CBD injury can be performed using three principally different techniques: direct suture repair or bilioenteric anastomosis with either a HD or hepaticojejunostomy Roux-en-Y (HJRY). For complete transection injuries of the bile duct most surgeons prefer HJRY as salvage therapy [2, 11, 12]. In the present case we preferred HD rather than HJRY. The reason for this was dual. Firstly, we found it important not to interfere with the Roux-en-Y loop already constructed to treat the morbid obesity. The weight loss this patient had achieved was satisfactory and the construction of a new Roux-en-loop for HJ was not attractive since there was a possibility of jeopardizing the result of the bariatric surgery. Secondly, data from liver transplant patients and experience from our transplant center have shown that HD gives satisfactory results [13, 14]. Others have found excellent results with HD that are comparable with HJRY in a variety of indications such as benign bile duct strictures, choledochal cysts, HPB-cancer, and bile duct injuries [15–18]. A recent report advocated HD as the preferred method of biliary reconstruction and described it is as a safe and simple technique with low rates of leak, stricture, cholangitis, and bile gastritis [17]. It is however important that the anastomosis is tension-free and Kocher's maneuver should be performed if necessary.

## 4. Conclusions

The present case report demonstrates the management of a bile duct injury induced by laparoscopic cholecystectomy following RYGB. The treatment of such bile duct injuries may represent a new challenge for HPB-surgeons and we suggest that surgery should be planned together with bariatric surgeons to prevent interference with previous surgery. We preferred to preserve the RYGB anatomy and to perform the biliary reconstruction end-to-side HD. There were no complications and the procedure did not affect the weight loss outcome of the RYGB. However, it will be interesting and useful to collect data pointing out eventual complications.
